# Remote sensing‐based landscape indicators for the evaluation of threatened‐bird habitats in a tropical forest

**DOI:** 10.1002/ece3.2970

**Published:** 2017-05-18

**Authors:** Minerva Singh, Timo Tokola, Zhengyang Hou, Claudia Notarnicola

**Affiliations:** ^1^ University of CambridgeCambridgeUK; ^2^ School of Forest SciencesUniversity of Eastern FinlandJoensuuFinland; ^3^ Department of Geography and Geographical Information ScienceUniversity of Illinois at Urbana‐ChampaignChampaignILUSA; ^4^ EURAC‐Institute for Applied Remote SensingBolzanoItaly

**Keywords:** forest patches, habitat suitability, IUCN Red listed birds, Laos, production forest, remote sensing

## Abstract

Avian species persistence in a forest patch is strongly related to the degree of isolation and size of a forest patch and the vegetation structure within a patch and its matrix are important predictors of bird habitat suitability. A combination of space‐borne optical (Landsat), ALOS‐PALSAR (radar), and airborne Light Detection and Ranging (LiDAR) data was used for assessing variation in forest structure across forest patches that had undergone different levels of forest degradation in a logged forest—agricultural landscape in Southern Laos. The efficacy of different remote sensing (RS) data sources in distinguishing forest patches that had different seizes, configurations, and vegetation structure was examined. These data were found to be sensitive to the varying levels of degradation of the different patch categories. Additionally, the role of local scale forest structure variables (characterized using the different RS data and patch area) and landscape variables (characterized by distance from different forest patches) in influencing habitat preferences of International Union for Conservation of Nature (IUCN) Red listed birds found in the study area was examined. A machine learning algorithm, MaxEnt, was used in conjunction with these data and field collected geographical locations of the avian species to identify the factors influencing habitat preference of the different bird species and their suitable habitats. Results show that distance from different forest patches played a more important role in influencing habitat suitability for the different avian species than local scale factors related to vegetation structure and health. In addition to distance from forest patches, LiDAR‐derived forest structure and Landsat‐derived spectral variables were important determinants of avian habitat preference. The models derived using MaxEnt were used to create an overall habitat suitability map (HSM) which mapped the most suitable habitat patches for sustaining all the avian species. This work also provides insight that retention of forest patches, including degraded and isolated forest patches in addition to large contiguous forest patches, can facilitate bird species retention within tropical agricultural landscapes. It also demonstrates the effective use of RS data in distinguishing between forests that have undergone varying levels of degradation and identifying the habitat preferences of different bird species. Practical conservation management planning endeavors can use such data for both landscape scale monitoring and habitat mapping.

## 1 INTRODUCTION

Remote sensing (RS) data have been widely applied for studying forest structure and its impact on bird species persistence (Eldegard et al., 2014). Optical imagery from Landsat has often been used to estimate vegetation properties such as greenness and its influence on avian persistence (Ranganathan, Krishnaswamy, & Anand, 2010). Landsat‐derived variables, such as greenness, allow us to quantify how vegetation properties vary with different levels of degradation. This makes them useful for monitoring forest transformation and degradation, including varying stages of forest degradation in human modified tropical ecosystems (Cattau, 2010; Wiens et al., 2009).

In addition to optical imagery, airborne Light Detection and Ranging (LiDAR) is another method used for monitoring forest structure dynamics and their impact on species. Although the use of LiDAR has been known to be costly (Hummel, Hudak, Uebler, Falkowski, & Megown, 2011), LiDAR provides more detailed information on forest structure such as tree heights (Shimizu et al., 2014). The three‐dimensional vegetation structure has been mapped using LiDAR data and was found to be associated with the presence and persistence of animal species in a given ecosystem (Davies & Asner, 2014). Moreover, LiDAR‐based measures of canopy structure and complexity were shown to be key predictors of the breeding habitat of a migrant Neotropical bird species in the United States (Goetz et al., 2010). Space‐borne radar provides another source of forest structural information at a global scale. A Radar Forest Degradation Index (RFDI) derived from these data was successful at detecting degraded forests (Mitchard et al., 2011). However, RFDI as yet has not been used to quantify avian habitat preferences in tropical forests. This study has used LiDAR, Landsat, and ALOS‐PALSAR RFDI to quantify their ability to distinguish between forest patches of different sizes that have undergone varying levels of degradation in a tropical forest–agricultural ecosystem in southern Laos. These RS variables were used in conjunction with a landscape level variable (distance from forest patches) to identify suitable habitat for endangered avian species. The study aimed to demonstrate the efficacy of different RS data and tools to monitor the inert‐related problems of forest degradation and habitat identification for endangered species is currently facing with the view of contributing to practical conservation management.

Southeast Asia's biodiversity faces an impending crisis. Over the past several decades, the region has the highest rates of deforestation in the world and it could lose three quarters of its forests by 2100 (Sodhi, Koh, Brook, & Ng, 2004). An analysis using a 500‐m resolution MODIS dataset from 2000 to 2011 in the Lower Mekong region revealed that Vietnam lost 1.1% of forests per annum, followed by Cambodia at 0.7% and Laos at 0.4% (Leinenkugel, Wolters, Oppelt, & Kuenzer, 2015). Such a rapid rate of deforestation could result in a large‐scale extinction of different species, including avian species. Given the high level of endemic species in the region, regional losses could translate into global extinctions (Sodhi et al., 2004).

The causes of forest loss vary across SE Asia. One of the leading causes of tropical forest degradation and biodiversity loss is selective logging; the practice of removing high value timber species. It has been considered as a more sustainable alternative to clear felling wherein all trees in the area are cut uniformly. Owing to high habitat specificity, many avian guilds need large patches of contiguous and near intact forests, and so decline in selectively logged forests (Tobias, Şekercioğlu, & Vargas, 2013). A meta‐analysis of the response of tropical and subtropical bird species to anthropogenic disturbances revealed that these species were generally less likely to occur in logged/disturbed forests (Newbold et al., 2014). Frugivorous birds are particularly affected by selective logging and higher logging intensities (Tobias, 2015). However, species richness of birds in intact primary forest, once‐logged and twice‐logged forest in Borneo were found to be similar and the International Union for Conservation of Nature (IUCN) threatened birds (listed as Near‐Threatened, Vulnerable or Endangered species) did not decline after the second round of logging. Hence, it is important to examine the ability of degraded and human‐modified tropical ecosystems to provide habitat to endangered avian species.

This study explores the use of space‐borne optical, radar, and airborne LiDAR datasets to quantify forest structural properties and to model the distribution of bird species in a human‐modified tropical landscape in Laos, the two conservation issues that concern the wider tropical Asia as well. We envisage that a combination of RS variables is needed to characterize the different properties of the human‐modified landscapes and their effect on habitat suitability of different bird species.

In addition to local scale factors (characterized by RS derived vegetation parameters and patch area), landscape scale factors in form of distance from forest patches have been included as this may influence species persistence in an agricultural landscape (Angelieri, Adams‐Hosking, de Barros, de Souza, & McAlpine, 2016). Using these inputs, we developed a combined habitat suitability map (HSM) for the bird species under consideration using a MaxEnt (maximum entropy) modeling approach (Elith et al., 2011). It has been suggested that landscape scale factors play an important role in influencing avian habitat preferences in temperate forests (Zhao, Azeria, Le Blanc, Lemaître, & Fortin, 2013). Landscape scale factors have been identified as being significant drivers of avian species habitat preferences in anthropogenically disturbed landscapes (De Barros, de Siqueira, Alexandrino, Da Luz, & Do Couto, 2012) and are known to be more important than local scale forest structure variables (Zhao et al., 2013). These findings make it important to quantify the role of both local and landscape scale factors in influencing avian habitat preferences in human‐modified tropical ecosystems such as those found in Laos and elsewhere in SE Asia. Evaluation of factors that influence habitat preference and identifying suitable habitat patches is important for practical conservation management. This research has the following objectives: (a) Evaluate how RS metrics pertaining to forest structure and degradation vary across forest patches that have undergone varying levels of degradation; (b) identify the most important local scale factor that contribute to habitat preferences of endangered birds; and (c) examine the role of a landscape level factor: distance from different patch types in influencing habitat suitability. It is expected that the results of (a) will help identify the extent to which different RS data can distinguish forest patches that have undergone varying levels of degradation and if they concur with the qualitative categorizations made on the ground. Further, (b) and (c) will help quantify the role of both local and landscape scale factors in influencing the habitat preferences of different avian species and help identify suitable habitat patches.

## 2 MATERIALS AND METHODS

### 2.1 Description of study site and bird surveys

**Figure 1:**
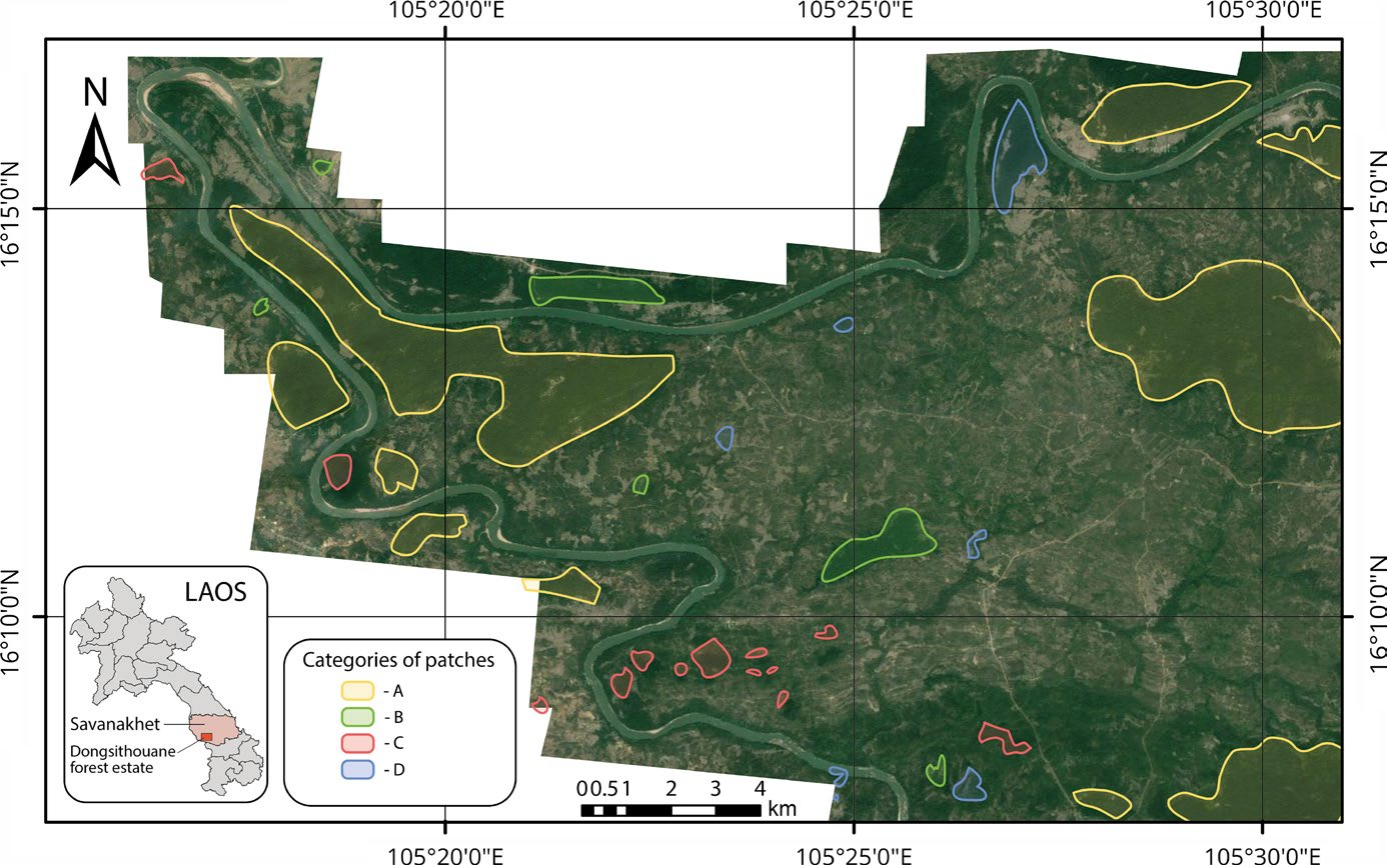
Dongsithouane production forest area located in Savannakhet Province of Laos (With Different Patch categories). Image © 2015 DigitalGlobe

Our study site is a 25,000‐ha estate called Dongsithouane (Figure 1), located in the Savannakhet province of southern Laos. Dongsithouane contains a mosaic of different land use types including natural closed canopy forests, open and highly degraded woodlands, scrublands, paddy fields, and human settlements. The forested areas are set aside for sustainable timber management and for supporting people's livelihood (The REDD Desk, 2016). The estate lies close to several IUCN VI protected areas, including Phou Xieng Thong, Dong Hua Sao and Ramsar wetland Xe Champone.

The area is a landscape of agricultural fields and patches of tropical woodland of varying sizes, configurations, and disturbance histories. Some of the patches continue to be exploited for timber. These patches have been categorized into different categories (A–D) on the basis of size, configuration, and the qualitative assessment of forest degradation. Local experts were employed to locate different forest patches and help categorize them qualitatively based on their structure, configuration/size, and magnitude of degradation and intactness. Using qualitative forest quality assessment indicators previously used by Turner et al. (2012), we examined if the forest patches had a closed canopy with tall trees or not. The presence of other hallmarks of disturbance and degradation such as the presence of vines, understory, and scrub was visually assessed as well. Based on these criteria, the different forest patch categories identified as a part of this research are: (A) large patches of closed‐canopy forest. These had tall trees, sparse understory, and no evidence of logging detected (mean size: 4.15 km^2^); (B) small forest patches with large canopy gaps, thick understory, and presence of many small trees. These were mostly found in the vicinity of villages (mean size: 0.38 km^2^); (C) small patches of closed canopy forest. The presence of some understory and several small trees was detected in these. These were located within woodlands or scrublands that had been heavily exploited for timber (mean size: 0.11 km^2^); and (D) highly degraded forest patches with an open canopy and scrub development (mean size: 0.31 km^2^). These different forest patches were then manually digitized on Google Earth.

Bird surveys were carried out in December 2015 with the assistance of a local forestry official in at least three patches from each of the categories, spanning as much of the production forest as possible, using a point count method. This method is useful for getting a spatially representative sample from each patch (Whitworth, Newman, Mundkur, & Harris, 2007). The surveys were carried out from 6 to 10 a.m. every morning with 200‐m interval between each of the points, to avoid double counting (dos Anjos et al., 2015; Mollon, 2010). This was performed to ensure we obtain spatially well distributed geo‐location data. About 15 min were spent at each point observing bird species, and birds seen or heard within a 20‐m vicinity of the observation point were recorded. Laos has a problem of unexploded landmines from the wars of 1960s and 1970s (Lao Rehabilitation Foundation, 2010). The impact on the study area is not known but the suspected presence of landmines has been given high attention so as not to impede data collection activities. The bird species presence data was recorded for IUCN Red listed threatened bird species observed in the area (Table 1). A map showing the geo‐locations of the different avian species has been provided in Figure S11.

**Table 1:** IUCN Red listed threatened bird species

IUCN Red listed threatened bird species	IUCN status
Green Peafowl (*Pavo muticus*)	Endangered (EN)
Ashy Headed Green Pigeon (*Trenon phayrei*)	Near Threatened (NT)
Alexander Parakeet (*Psittacula eupatria*)	Near Threatened (NT)
Blossom headed Parakeet (*Psittacula roseata*)	Near Threatened (NT)
Asian Golden Weaver (*Ploceus hypoxanthus*)	Near Threatened (NT)
White‐Rumped Pygmy Falcon (*P. insignis*)	Near Threatened (NT)
Red Collared Woodpecker (*Picus rabieri*)	Near Threatened (NT)

Seven Red listed bird species are investigated in this work. The birds under consideration use different parts of the landscape for fulfilling different life functions. The species under consideration (Table 1) inhabit woodlands and forests but also use the surrounding agricultural matrix for feeding and transverse the landscape. For instance, parakeets are known to inhabit a variety of forested habitats but also forage in agricultural areas for food (Thewlis, Timmins, Evans, & Duckworth, 1998). Same for the endangered green peafowl which inhabits a variety of forest habitats and can also use agricultural edges for foraging. This was observed during the course of the fieldwork as well. The ability of mobile taxa to use different parts of the forest for different life functions has been observed in the case of other mobile taxa as well (Morrogh‐Bernard, Husson, Harsanto, & Chivers, 2014). Hence, in a human‐modified tropical forest ecosystem, it is vital we identify the local and landscape scale factors that may influence avian species persistence.

### 2.2 Airborne LiDAR survey

The airborne LiDAR data were obtained in February 2009, using a Piper PA‐31 Najavo aircraft at an altitude of 2,000 m and a flying speed on 19 knots. Leica Airborne Laser Scanner (ALS) was used with a 30‐m field of view (FOV), delivering a point density of 1 point/m^2^ and two returns (first and last) only. TerraScan was used for data preprocessing and initial point classification (Hou, Xu, & Tokola, 2011). Area‐based LiDAR metrics were derived using LAS tools (Isenburg, M., 2014) for the vertical and horizontal vegetation structure using a 20 m by 20 m (see Table 2). Vertical distribution ratio (VDR) also helps quantify the subcanopy variation in the tree canopies (Hill & Hinsley, 2015).

**Table 2:** Area‐based LiDAR metrics and their ecological significance

Name of the LiDAR metric	Ecological significance	References
Max. height	2D representation of the tallest trees in the landscape.	
Average height	2D representation of the average tree height in the landscape.	
Height percentiles (p10, p20, p50, p90)	Captures the percentile distribution of laser returns within the canopy. Height percentiles were calculated from 5% to 95% with the view of representing the multilayered structure of the forest canopy.	Laurin, Chen, Arthur, and Valentini (2014), Wasser, Day, Chasmer, and Taylor (2013), Garcia, Riaño, Chuvieco, and Danson (2010)
Skewness	Value increases with increased canopy height and development. Hence, it is a measure of both canopy complexity and vertical distribution.	Ediriweera (2013)
Standard deviation	Represents variation in canopy structure	Isenburg (2014)
Canopy cover	Number of first returns above a given height cutoff is divided by the total number of first returns. It measures the extent of ground covered with vegetation.	Davies and Asner (2014), Isenburg (2014)
Vertical distribution ratio (VDR)	Vertical distribution ratio (VDR) quantifies the vertical distribution of foliage in the canopy. Forest patches with a closed canopy and reduced understory have a low VDR	Goetz, Steinberg, Dubayah, and Blair (2007)

The avian geo‐location data were collected in 2015. The forest change is assumed to be low as no forest clearance activity has occurred in the study area; hence, it is appropriate to compare these data. Earlier works suggest that a time lag of 6 years or more between LiDAR and field data collection has no impact on patterns of avian distribution in relatively undisturbed forests (Hill & Hinsley, 2015; Vierling, Swift, Hudak, Vogeler, & Vierling, 2014).

### 2.3 Radar imagery (ALOS‐PALSAR)

ALOS‐PALSAR data collected in 2010 with a spatial resolution of 25 m were downloaded from the JAXA website (JAXA, 2010). These were provided in a color‐composite form and had dual polarization HH (horizontal transmit, horizontal receive) and HV (horizontal transmit, vertical receive). These data are provided as digital numbers (DN). In order to obtain the backscatter values, the DN values of both HH and HV were converted to the normalized radar cross section (σ^0^) as follows (Avtar, Suzuki, Takeuchi, & Sawada, 2013):

(1) $$\sigma^{0}=10\cdot \log_{10}\left({\text{DN}}^{2}\right)+\text{CF}$$

Here, CF is a calibration factor (−83 in case of ALOS‐PALSAR data). An enhanced Lee filter was applied to reduce speckle, while retaining the image texture using ENVI. HH and HV values were used to calculate the Radar Forest Degradation Index (RFDI), a radar‐derived measure of forest degradation (Mitchard et al., 2011):

(2) $$\text{RFDI}=\frac{\text{HH}-\text{HV}}{\text{HH}+\text{HV}}$$

The backscatter values are strongly associated with the forest structural components, orientation, and factors such as canopy cover (Mitchard et al., 2011). Hence, when the canopy opens up (as a consequence of logging), this causes RFDI to go up. Completely cleared areas have RFDI value of 1, while undisturbed forests have RFDI values ranging from 0.3 to 0.4 (Saatchi, 2007).

### 2.4 Landsat

Landsat‐5 data collected in May 2010 were downloaded from the Landsat/USGS website (U.S. Geological Survey, 2016; USGS, 2015). ClasLite was used for radiometric and atmospheric correction of the raw Landsat images, in order to derive top of the surface reflectance for all the spectral bands (Asner, Knapp, Balaji, & Páez‐Acosta, 2009). ClasLite implements sensor‐specific offset and gains to convert the raw DN values to radiance (expressed in terms of watts/m^2^/solid angle). These were in turn put through the 6S atmospheric correction module provided by ClasLite to produce top of the atmosphere surface reflectance (Bryan et al., 2013). Tasselled cap transformation was implemented on these atmospherically corrected surface reflectance data. A tasselled‐cap transformation is a technique that converts the original bands of Landsat data (blue, green, red, NIR, SWIR1, SWIR2) into a new set of variables (Kauth & Thomas, 1976). Tasselled cap transformations of the reflectance data (using ENVI) reduced data from seven to three ecologically significant bands (i.e., brightness, greenness, and wetness) (Ranganathan et al., 2010). Brightness is a measure of soil brightness, greenness is associated with chlorophyll content and canopy density, while wetness is related to water content of the vegetation (Foster, Townsend, & Zganjar, 2008).

Kruskal–Wallis tests were carried out to examine how the vegetation properties, derived using LiDAR, Landsat, and ALOS PALSAR, varies among the four patch categories. Using the “pgirmess” package in R, post hoc analysis was conducted to identify which patches were significantly different.

### 2.5 Species and habitat suitability maps using MaxEnt

MaxEnt is a machine learning‐based technique which uses species presence data in conjunction with environmental layers for modeling species distribution (Elith et al., 2011). The algorithm seeks to find the distribution of maximum entropy that agrees with the value of environmental layers where species presence has been recorded (Lahoz‐Monfort, Guillera‐Arroita, Milner‐Gulland, Young, & Nicholson, 2010). This algorithm works with species presence data and can be used with a small sample size (Norris, 2014; Tinoco, Astudillo, Latta, & Graham, 2009). The inability to collect absence data is a common problem in many wildlife surveys. MaxEnt essentially works as a presence‐pseudoabsence model by making use of specified number of background points (in this case 10,000) along with the presence data collected in field (Redon & Luque 2010). The previously described RS variables were used as environmental layers (Huang et al. 2014). These variables represent the local scale forest structure‐related variables that influence habitat suitability. Additionally, Euclidean distances to the four different patch categories (A, B, C, and D) were included as landscape level variables. In its rasterized format, the value of each pixel is its Euclidean distance from its nearest forest patch edge. Two separate models were developed using MaxEnt: one with a combination of local and landscape scale variables, and another one with local scale variables only. It was expected that the former will help us understand the role distance from forest patches can play in influencing habitat preference in a mixed ecosystem like ours, and the latter is expected to help identify the local scale factors that influence habitat suitability.

The modeling was performed using MaxEnt software (version 3.3.k) (Princeton, 2015). Regularization was implemented to penalizing the model in proportion to the coefficient magnitude, thus preventing overfitting (Merow, Smith, & Silander, 2013). Further, 1,000 iterations were carried out to allow for convergence, thereby reducing the chance of over or underprediction (Frake, 2014). Variable importance estimation was carried out using both the heuristic test and the jackknife procedure; the latter helps to evaluate the independent contribution of variables to the habitat suitability model (Padalia, Srivastava, & Kushwaha, 2014). It should be noted that 75% of the training data were used for model training and 25% data were employed for model testing (Lahoz‐Monfort et al., 2010). The area under the curve (AUC) was also computed for evaluating model performance using the test data. The AUC of a model is a threshold independent measure equivalent to the probability that the model will rank a randomly chosen species presence site higher than a randomly chosen absence site (Liu, White, & Newell, 2011).

The species distribution models with AUC ≥ 0.7 were retained for further analysis. Models with AUC ≥ 0.7 are regarded as useful in the literature, and those <0.7 are deemed underperforming thus discarded (Hoffman, Narumalani, Mishra, Merani, & Zilson, 2008; Pearson, Raxworthy, Nakamura, & Peterson, 2008; Reside, VanDerWal, & Kutt, 2012). The model produces a habitat suitability map for each species on 0–1 scale where 1 indicates the most suitable habitat and 0 least suitable habitat (Melin, Packalén, Matala, Mehtätalo, & Pusenius, 2013). Schoener's D index was computed from these outputs to assess the species habitat overlap (Chetan, Praveen, & Vasudeva, 2014). The species distribution models (SDMs) of individual species were summed to obtain a combined habitat suitability model (HSM) (Wordley, Sankaran, Mudappa, & Altringham, 2015). This was again scaled to 0–1. For mapping purposes, areas with values below 0.2 are designated as unsuitable, between 0.2 and 0.6 are marginally suitable and above 0.6 are highly suitable habitats (Nazeri et al., 2012). The purpose of this analysis was to identify the most suitable habitat patches (areas that have highest habitat suitability for all avian species combined).

## 3 RESULTS

### 3.1 Variation in vegetation structure across forest patches

**Figure 2:**
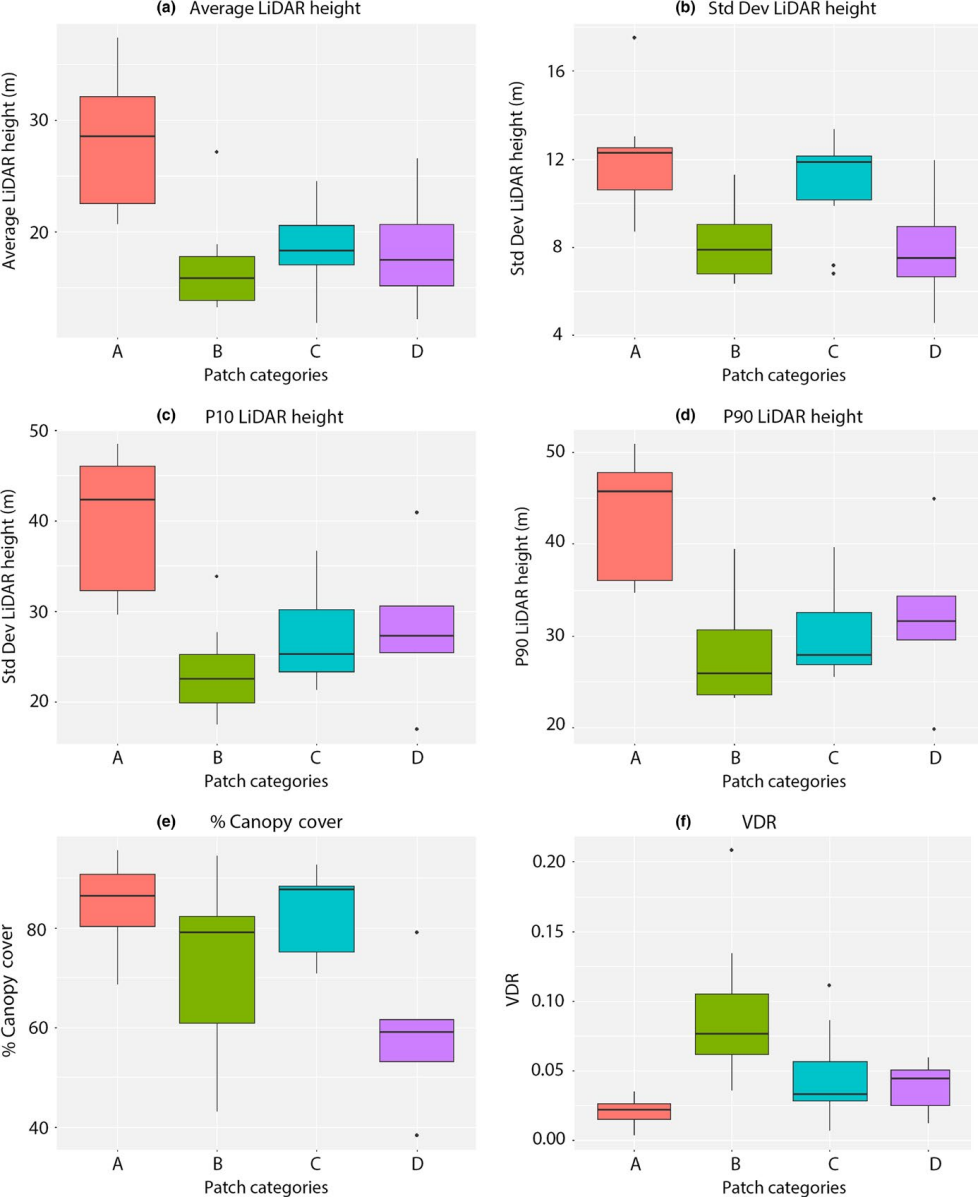
LiDAR measures of the (a) average LiDAR height, (b) standard deviation LiDAR height, (c) 10th LiDAR percentiles, (d) 90th LiDAR percentiles, (e) %canopy cover, and the (f) vertical distribution ratio (VDR) structures across different patch configurations for the forest patches in four categories: (A) large patches of closed‐canopy forest, (B) small forest patches with large canopy gaps, thick understory, and the presence of many small trees., (C) small patches of closed forest within a matrix of exploited woodland, (D) highly degraded forest patches with an open canopy and scrub development

Four patch types of varying sizes, spatial configuration, and degradation level categories (A–D) were evaluated as part of this research. Vertical vegetation parameters (derived from LiDAR) varied significantly among the four patch categories (Figure 2a,b).

The average height, as well as the standard deviation height differed among the four patch types (KW statistic = 17.92, 14.1, respectively, *p* < .01). The average canopy height was found to be significantly different across all four patch categories while the standard deviation of height only varied significantly between patch categories A and B. Large forest patches (A) had the highest median value of these variables. Large forest patches (A) also had significantly different values in all the percentile height bins compared with the smaller patches (B–D): p10 (KW = 17.66, *p* < .01) and p90 (KW = 16.23, *p* < .01) as shown in Figure 2c,d.

Large forest patches (A) and small patches within a matrix of degraded forests (C) had significantly higher canopy cover (KW = 10.43, *p* < .01), indicating greater canopy intactness and structural complexity compared with small isolated patches (B) and highly degraded forest patches (D) (Figure 2e,f).

**Figure 3:**
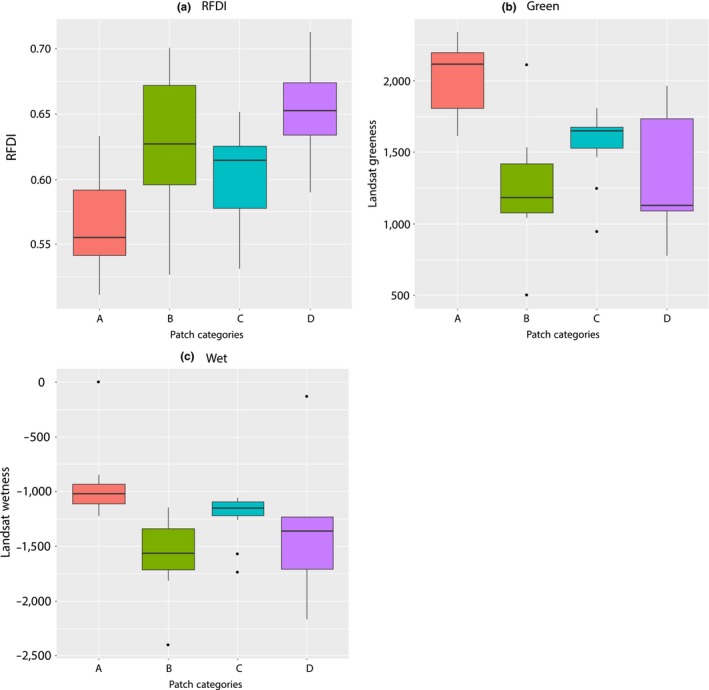
Variation in the (a) radar forest degradation index (RFDI), (b) greenness, and (c) wetness across different patch types: (A) large patches of closed‐canopy forest, (B) small forest patches with large canopy gaps, thick understory, and the presence of many small trees., (C) small patches of closed forest within a matrix of exploited woodland, (D) highly degraded forest patches with an open canopy and scrub development

The VDR was significantly lower for large forest patches (A) compared to the other forest patches (KW = 16.18, *p* < .01), indicating the presence of a relatively closed canopy structure and reduced understory compared to other patch configurations. Patch A had also a significantly lower ALOS PALSAR RFDI value compared with other forest patch classes, indicating low rate of forest degradation and anthropogenic disturbance (Figure 3a).

Large forest patches (A) and small close‐canopied forest patches (C) had lower values of RFDI than patch categories (B) and (D), indicating that they were less degraded (KW = 11, *p* < .01). Patch category A (followed by patch category C) has the highest values of greenness and wetness (Figure 3b,c).

Patch categories (A) and (C) have significantly higher values of Landsat‐derived wetness and greenness, indicating higher levels of photosynthetically active and low levels of degraded vegetation. Further the values of greenness varied significantly across all four patch categories and wetness varied significantly between all patch categories except between (A) and (C).

### 3.2 Species and habitat suitability mapping for avian species

Two MaxEnt models were developed. In the first model, only the local scale forest structure variables (derived from LiDAR, Landsat and ALOS‐PLASAR) were modeled. The AUC of SDMs for all species (except the blossom headed parakeet, green peafowl, and the white‐rumped pygmy falcon) was >0.7. The percentage contribution of each of the local scale factors in explaining habitat suitability of individual species is presented in Table 3.

**Table 3:** Percentage contributions of local scale factors to habitat suitability of different species

	Alexandrine parakeet	Ashy headed green pigeon	Asian golden weaver	Blossom headed parakeet	Green peafowl	Red collared wood‐pecker	White rumped pygmy falcon
Brightness	5.6	0	0.4	17.8	0	2	0
% Canopy cover	13.7	7.1	5.3	17.9	0	0.3	26.6
Greenness	8.3	1.4	0	10	80.3	0	1.1
Standard deviation of LiDAR height	3.6	0	0.5	3.5	0.7	46.5	0.8
Maximum LiDAR height	7.3	0	0.8	0	1.7	0	2.4
10th percentile heights	26.9	31	19.3	29.2	0	18.1	5.8
50th percentile heights	7.8	3.9	0.3	1.4	0	0	0.2
90th percentile heights	0.4	0	4.2	0.9	0	0	0
Radar forest degradation index	0.4	0.4	6.3	5.6	0	0	0.2
LiDAR height skewness	0	0	8.6	0.7	0	13.2	5.3
Vertical distribution ratio	18.9	1.2	4.4	1.4	0	9.3	43.2
Patch area	6.9	54.9	49.9	11.5	17.2	10.7	14.3
Total	100.0	100.0	100.0	100.0	100.0	100.0	100.0
Area under the curve	0.72	0.92	0.73	0.5	0.24	0.86	0.53

In addition to LiDAR structural variables, Landsat‐derived variables of canopy spectral properties such as greenness also influence habitat suitability for different avian species at local scale. Landsat‐derived spectral metrics are important explanatory variables along with LiDAR‐derived structural variables.

In the second model, we included both local scale forest structure variables and landscape factors (Euclidean distances between bird species presence and the four different forest patches). The AUC of all the SDMs (except for green peafowl and red collared woodpecker) was >0.7. For all the cases with AUC > 0.7, the landscape scale factors (distance from different forest patches) had higher percentage contribution to habitat suitability models of individual bird species than local scale factors (Table 4).

**Table 4:** Percentage contribution of both local scale and landscape scale factors/distance of forest patches, where (A) large patches of closed‐canopy forest, (B) small forest patches with large canopy gaps, thick understory, and the presence of many small trees., (C) small patches of closed forest within a matrix of exploited woodland, (D) highly degraded forest patches with an open canopy and scrub development

	Alexandrine parakeet	Ashy headed green pigeon	Asian golden weaver	Blossom headed parakeet	Green peafowl	Red collared woodpecker	White rumped pygmy falcon
Brightness	0	0.1	1.1	0	0	0	0.9
% Canopy cover	13.5	1.7	2	6.1	34.9	0	3.1
Greenness	0.8	0.1	0	0	1.4	0	0
Standard deviation of LiDAR height	6.7	0	0.4	0	6.1	9.4	0.4
Maximum LiDAR height	0	0.5	0	0.8	0	0	0.7
10th percentile heights	7.2	11.9	0	1	0	17.5	0.9
50th percentile heights	1	0	0.3	0	0	0	0
90th percentile heights	0.2	0.8	0.5	0.1	0	0	0
Distance from patch A	2.5	70.1	20.9	1.3	0	0	13.1
Distance from patch B	42.6	0	4.6	78.6	57.5	0	17.6
Distance from patch C	0.2	0	16.4	4.9	0	4.5	10.5
Distance from patch D	18.7	0	50	6.6	0	6.8	27.3
Patch area	0.5	11	1.4	0	0	0.8	14.3
Radar forest degradation index	1	1.7	0	0	0	7.5	0
LiDAR height skewness	0	0.2	1.3	0.4	0	20.3	2.2
Vertical distribution ratio	5.1	1.7	0.1	0	0	33.2	9
Total	100.0	100.0	100.0	100.0	100.0	100.0	100.0
Area under the curve	0.94	0.95	0.97	0.72	0.53	0.68	0.93

The species habitats were also found to moderately overlap with Schoener's D index ranging from 0.1 to <0.7 (Table 5). A D index of 0.8 and above would indicate a significant overlap of species habitats.

**Table 5:** D‐index of avian species overlap

	White rumped pygmy falcon	Blossom headed parakeet	Asian golden weaver	Ashy headed green pigeon	Alexandrine parakeet
White rumped pygmy falcon					
Blossom headed parakeet	0.565				
Asian golden weaver	0.603	0.435			
Ashy headed green pigeon	0.318	0.389	0.175		
Alexandrine parakeet	0.518	0.612	0.415	0.4	

Jackknife test of variable importance also revealed that distance of different patches had more information by themselves as compared to local forest structure variables (see Figures S1–S10). The individual SDMs produced by this model (also presented in the Figures S1–S10) with test AUC values >0.7 were retained for producing a cumulative HSM, as shown in Figure 4.

**Figure 4:**
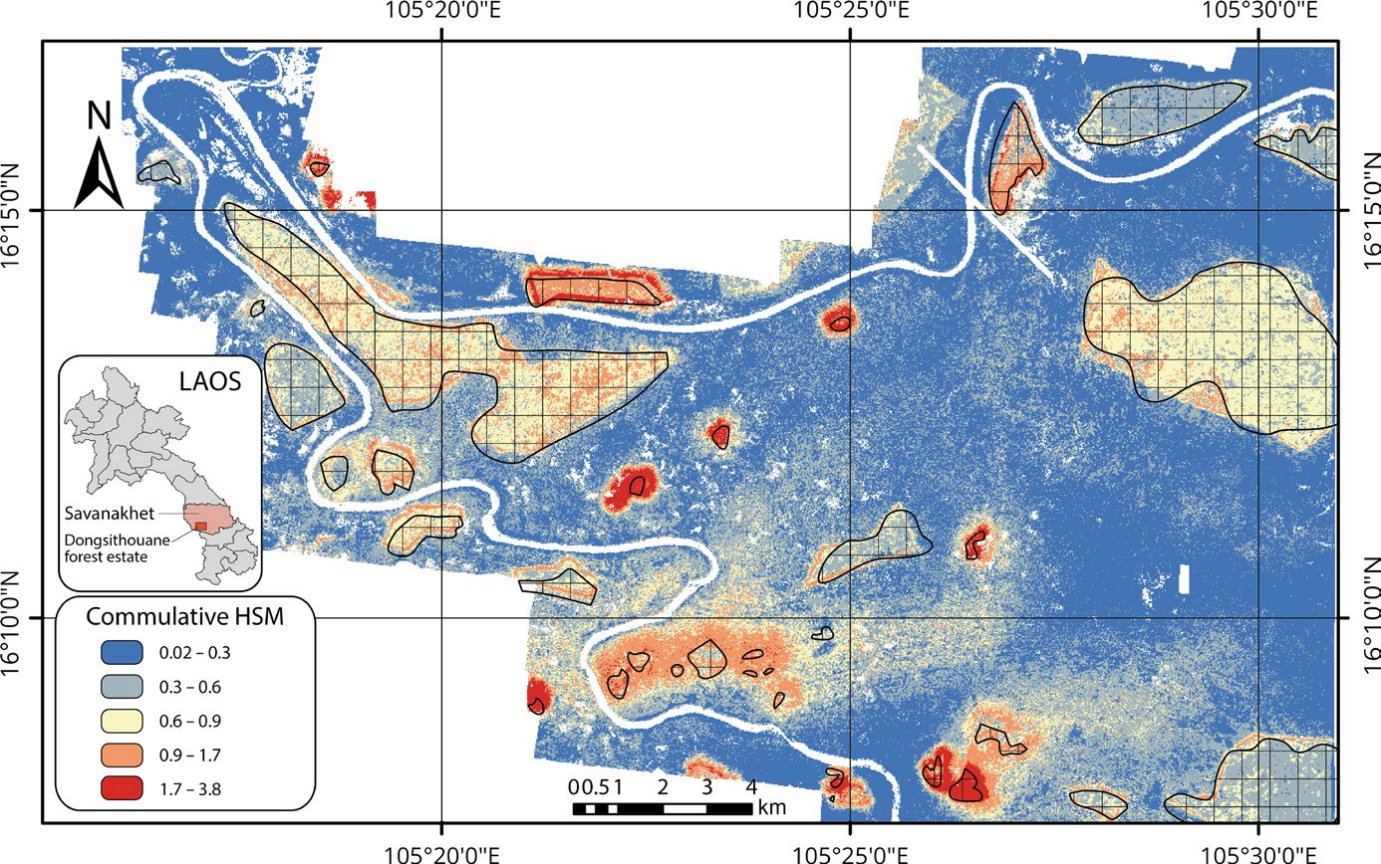
Cumulative habitat suitability map (HSM) for all avian species under consideration

The cumulative HSM was normalized, and three categories were computed: (1) unsuitable, (2) marginal habitat, and (3) highly suitable habitat using the thresholding scheme recommended by Nazeri et al. (2012) Analysis of this scaled HSM revealed that around 1% of the study area was highly suitable for all avian species under consideration. However, the percentage availability of suitable and marginal habitats varied considerably for individual bird species (Table 6).

**Table 6:** Suitable habitats for individual bird species (%)

	% Unsuitable habitat	% Marginal habitat	% Highly suitable habitat
Alexandrine parakeet	91.55	7.26	1.18
Ashy headed green pigeon	81.76	12.41	5.83
Asian golden weaver	93.24	6.05	0.72
Blossom headed parakeet	94.41	4.55	1.04
White rumped pygmy falcon	86.32	11.6	2.08

## 4 DISCUSSION

This study had two overarching aims. First was to evaluate the ability of different RS metrics to distinguish between forest patches that have faced different levels of degradation and have different sizes and configurations. Second was to identify suitable habitats for avian species in a tropical forest–agricultural ecosystem. Section 4.1 discusses the findings pertaining to the former while Sections 4.2 and 4.3 discuss the latter.

### 4.1 Variation in forest structure across different patch types

Large forest patches were distinctly different in structure to the small patches. The tallest forest was found in the large forest patches (A, median height = 18 m). A detailed analysis of tree species within the different patch types is outside the scope of this research, but it may be inferred that these large patches still retains tall Dipterocarp tree species, which once dominated much of the lowland ecosystem in the Greater Mekong region and can reach heights of up to 40 m (Benders‐Hyde, 2002). The large patches also had the highest % canopy cover. These results are consistent with those of Flaspohler et al. (2010), who showed that patch size is strongly associated with canopy height 1. Compared with large patches, patches B and C had a high variability in the values of greenness and wetness, reflecting greater structural heterogeneity within these smaller patch types. In addition to the LiDAR‐ and Landsat‐derived metrics, RFDI varied across forest patches of different sizes. Based on this research and that of Mitchard et al. (2011), RFDI is a useful metric for quantifying changes in forest degradation.

Our research has demonstrated that data from multiple sources can be used to quantify the magnitude of degradation in forest patches. Further, the some of the RS metrics evaluated in this study (such as average forest canopy height, Landsat‐derived greenness) varied significantly across all four patch categories. The patch categories were determined on the basis of qualitative field assessments carried out by local experts. These findings indicate that derived qualitative assessments too can help in characterizing forest and other land use types. This has potential conservation ramifications. Conventionally, forest types in tropical Asia are classified on the basis of ecological classifications such as those suggested by Whitmore (Whitmore, 1975). However, these classifications do not account for the land use history of forests and different magnitudes of degradation. As tropical Asia continues to be dominated by human‐modified landscapes, it is important to identify and differentiate forests that have undergone varying levels of degradation and incorporate local land use categorizations into the conservation management endeavors. The findings of this research can further inform other aspects of forest functioning such as magnitude of forest fragmentation and isolation. Combined data from different sources can be used to provide a rapid large‐scale assessment of forest structural and spectral properties across human modified tropical landscapes particularly relevant in post‐war regions where the presence of landmines severely impairs the possibility of detailed field studies.

### 4.2 Factors affecting bird species habitat suitability

Several factors have been identified by MaxEnt modeling as influencing IUCN Red listed threatened bird species, including LiDAR‐derived variables of vertical and horizontal canopy structure. Research by Tattoni, Rizzolli, and Pedrini (2012) indicates that inclusion of LiDAR variables improves the predictive ability of MaxEnt‐based habitat suitability models (Tattoni et al., 2012). Variables related to forest structural variability are also important contributors to habitat suitability for species such as the ashy headed green pigeon and red‐collared woodpecker (especially when distance to forest patches is not considered). Studies by Flaspohler et al. (2010) and Jung, Kaiser, Böhm, Nieschulze, and Kalko (2012) indicate that higher structural heterogeneity can facilitate the persistence of multiple species even in human‐modified forest ecosystems. In case of certain species such as the ashy headed green pigeon, alexandrine parakeet, and blossom‐headed parakeet, the tasselled cap metrics were found to contain some useful information. Certain tasselled cap metrics such as wetness are strongly linked with the landscape scale canopy cover and the persistence of bird species in forested landscapes (Ranganathan et al., 2010) and arboreal mammal species (Turner et al., 2012). These metrics are important determinants of richness of forest dependent bird species and allow for finer distinction between different habitats as compared to measures such as canopy cover (Ranganathan, Chan, & Daily, 2007). These findings are further corroborated by Eldegard et al. (2014) who discovered that inclusion of spectral variables along with LiDAR improved the performance of models predicting avian species persistence in a boreal forest. Further, the size of patches was discovered to be an important variable for influencing habitat suitability. It has been discovered that in addition to forest structure within the patch, forest patch size is an important determinant of habitat preference, especially for species such as the ashy headed green pigeon and white rumped pygmy falcon. This finding is consistent with a previous study (Tobias et al., 2013) which indicates that patch area can influence the persistence of certain species (Davies & Asner, 2014). To the best of our knowledge, RFDI has not been previously employed to study avian habitat preferences. While RFDI is sensitive to variations in forest degradation across different forest patches, its contribution to explaining avian habitat preference was minimal.

The research has identified that different bird species have different local habitat structure preferences and limited habitat overlap (Whitmore, 1975). This information indicates different habitat preferences of the different avian species and can help customize conservation programs to suit the needs of the bird species in question. When landscape and local scale variables were modeled together, the former were discovered to be the more important variables for habitat suitability in almost all the cases. This is consistent with the existing body of research. Distance from forest patches was an important determinant avian persistence in the fragmented woodlands of England (Hinsley, Bellamy, Newton, & Sparks, 1995). It has further been established that mobile taxa such as mammals and birds can use agricultural landscapes for feeding and dispersal, but need forest patches for their continued persistence (Anand, Krishnaswamy, & Das, 2008; Angelieri et al., 2016). Indeed, in the study area, different avian species were observed foraging in the agricultural fields. However, this research has established the importance of both forest patch and landscape variables in facilitating avian species persistence and identified which of these are the most important for different species persistence in a tropical forest–agricultural matrix.

The presence of a contiguous forest cover is an important determinant of bird assemblages (Graham & Blake, 2001). In this research, distance from the large patches was found to be very important for the ashy headed green pigeon. Additionally, varying levels of canopy closure are also known to support bird species diversity (Gil‐Tena, Saura, & Brotons, 2007). This arguably explains why the distance from moderately disturbed, noncontinuous forest patches are an important variable of habitat suitability for many bird species such as the alexandrine parakeet and the blossom headed parakeet. These findings have important conservation ramifications. The role of logged forests, including twice logged forests can play in retention of IUCN Red listed imperilled birds in Borneo, has been established by Edwards et al. (2011). The findings of our research also make a case for retaining forest patches, including degraded forest patches within an agricultural ecosystem for bird species conservation. The authors infer that degraded forests serve as a vital habitat for the IUCN Red listed species (Edwards et al., 2011).

Our research has also demonstrated that MaxEnt is a suitable tool for modeling distribution of multiple species (and identifying factors that affect habitat suitability) using a combination of field collected data, multiple RS data for characterizing forest structure, and landscape variables. Indeed, MaxEnt has been used for modeling species distribution and identifying factors influencing habitat suitability in a number of tropical forest ecosystems (Lahoz‐Monfort et al., 2010; Nazeri et al., 2012; Wilting et al., 2010). Owing to its ability to produce useful results with a very small presence data, MaxEnt has proven itself to be useful for modeling the distribution of rare and endangered species (Rebelo & Jones, 2010; Tinoco et al., 2009). The ability to understand the role of both local scale forest structure and landscape scale variables and how they vary for different species can inform conservation management strategies on ground. Additionally with the use of different data sources, such models can help integrate the habitat requirements of individual species into land use and participatory forest management planning. A newly proposed framework for multispecies monitoring over a landscape scale builds on the concepts of systematic conservation planning (SCP) and recommends the building of SDMs can yield significant management benefits with lesser survey effort (Carvalho, Gonçalves, Guisan, & Honrado, 2015). The individual MaxEnt‐derived SDM stacking technique of habitat suitability determination employed in this research has been commonly used for conservation prioritization in other parts of the world. MaxEnt produced robust distribution models for threatened and endemic reptile species of Mexico and the stacked models derived subsequently were used for selecting important sites for conservation and setting up of future protected areas (Urbina‐Cardona & Flores‐Villela, 2010). While a detailed analysis of conservation prioritization in southern Laos is beyond the scope of this research, it is suggested that suitable habitats identified for a given set of species (using MaxEnt modeling and subsequent SDM stacking) be considered for future conservation area or protected area sites.

### 4.3 Avian persistence in human‐modified landscapes and proposed conservation action

As human pressure on natural ecosystems grows, it is important to evaluate the ability of the matrix surrounding nature reserves, agro‐ecosystems, human‐modified landscapes, agricultural fields, and plantations to provide habitat and feeding/foraging space to avian species (Petit, Petit, Christian, & Powell, 1999). The most suitable habitats are isolated patches located within a sea of marginal to unsuitable habitat matrix (see Figure 4). This is to be expected, given the magnitude of human modification of the landscape and conversion to paddy fields (Azman et al., 2011). Conversion to monoculture systems, such as rice paddies, brings about a decline in bird species abundance (Azman et al., 2011). Further, increased fragmentation and isolation of forest patches also leads to a decline in avian species abundances (Haddad et al., 2015). Rice paddy agricultural systems have been a part of tropical Asia's ecosystems for more than a millennium. The findings of this research indicate that retention of forest patches, including small degraded forest patches, could help with avian species retention within a rice monoculture‐dominated ecosystem.

Distance from the different forest patches is shown to have played an important role in influencing avian habitat preference. However, other local scale variables, notably, patch area was found to be an important determinant as well, along with variables (such as those derived from Landsat and LiDAR data) pertaining to forest structure and health (especially when the role of forest patch distance was excluded from the analysis). Research by Chang, Quan, and Wang (2013) and Flaspohler et al. (2010) indicates that smaller forest patches can play an important role in avian species conservation (see Table 4). The research has also identified that forest structure and spectral variables (derived from the LiDAR and Landsat data, respectively) are important for influencing individual avian species habitat preferences. The forest patches are isolated in a sea of unsuitable habitat (see Figure 4). This could increase the risk of “extinction debt,” the possibility of species going extinct after a time lag (Cowlishaw, 1999). However, ecological restoration of forest ecosystems is a stated goal of the Sustainable Forestry and Rural Development (SUFORD) program. Conservation action could focus on retaining and enhancing the forest structure and health variables that were identified in order to inform future ecological restoration programs, along with preserving the suitable habitats mapped. Restoration of marginal habitats can yield significant conservation benefits for avian species (Reid, Mendenhall, Rosales, Zahawi, & Holl, 2014).

Our research was conducted in a production forest in Laos. The tropical forest–agricultural matrix found here is a microcosm of tropical landscapes in the majority of the Greater Mekong region. Hence, it is important to devise strategies that can help with biodiversity retention in human‐modified landscapes and allow for landscape scale monitoring of forest degradation. The role large contiguous forest patches can play in bird species conservation has been well established, and it has been recommended that retention of large forest patches can help maintain a diverse species assemblage in human‐modified ecosystems (Fischer, Lindenmayer, & Manning, 2006). In addition to this, the research also makes a case for retaining small, degraded, isolated forest patches within agricultural landscapes. Previous research indicates that the presence of different kinds of forest patches helped improve the diversity of butterfly assemblages and small, isolated forest patches can contribute to landscape scale diversity (Benedick et al., 2006). Research by Struebig, Kingston, Zubaid, Mohd‐Adnan, and Rossiter (2008) suggests the conservation value of small degraded forest patches should not be ignored in human‐modified tropical ecosystems, and the presence of these can improve species dispersal across the landscape (Estrada & Coates‐Estrada, 2002).

Further, local scale factors influencing habitat suitability for different bird species and the availability of suitable habitat varies considerably between the different bird species under consideration. It is imperative future conservation actions focus on accounting for the habitat preference of individual avian species. Our research also indicates while LiDAR forest structure variables are important for explaining habitat preference, landscape scale factors (in this case distance from different forest patches), and Landsat‐derived variables are also useful indicators of habitat preference. Given that LiDAR data are expensive, future research could focus on using a combination of landscape scale variables along with Landsat‐derived metrics for identifying suitable habitats in areas that do not have LiDAR coverage.

## 5 CONCLUSION

This research has demonstrated how a combination of optical, radar, and LiDAR data may be used for assessing forest degradation and variation in forest structure across varying disturbance regimes/patch configurations. This is of potential importance in postconflict countries such as Laos which are rich in tropical forests. These countries face an ongoing problem of the presence of unexploded landmines and shells, which makes the collection of field data extremely hazardous. As this work demonstrates, RS data from different sources can be employed for rapid conservation monitoring by facilitating landscape scale identification of forest structure patterns and suitable habitats. Data from multiple sensors are useful for identifying the factors that influence avian habitat preference in a tropical forest–agricultural matrix ecosystem. Our findings make a case for promoting the retention of forest patches that includes degraded forest patches within an agricultural ecosystem for bird species conservation.

## CONFLICTS OF INTEREST

The authors have no conflicts of interest to declare.

## AUTHOR CONTRIBUTIONS

MS collected the field data, analyzed the data, and wrote the manuscript. TT, ZH, and CN provided suggestions on manuscript improvement. TT and ZH provided information about the study site and CN advised on ALOS PALSAR data processing
