# Supplementary material for: Remote sensing‐based landscape indicators for the evaluation of threatened‐bird habitats in a tropical forest
Source: Ecol Evol. 2017 May 18;7(13):4552–67. doi: 10.1002/ece3.2970 (PMC5496523; doi:10.1002/ece3.2970)
Supplement: Supplementary file 1 [file ECE3-7-4552-s001.docx]

**Remote sensing based landscape indicators for the evaluation of threatened-bird habitats in tropical forest**

Minerva Singh1* Timo Tokola^2^, Zhengyang Hou^3^, Claudia Notarnicola^4^

**Author Affiliations:**

^1^Department of Plant Sciences, Downing Street, University of Cambridge, Cambridge, CB2 3EA, UK

^2^University of Eastern Finland, School of Forest Sciences, P.O. Box 111, Fin-80101 Joensuu

^3^Department of Geography and Geographical Information Science, University of Illinois at Urbana-Champaign, 228 Computer Applications Building, MC-150 605, East Springfield Avenue, Champaign, IL 61820-6371, USA

^4^EURAC-Institute for Applied Remote Sensing, viale Druso 1, 39100 Bolzano, Italy

**SUPPLEMENTARY MATERIAL**

[Figure S1. Species distribution model for Alexandrine parakeet](#_Toc475040065)

[Figure S2. Jackknife of regularized training gain for Alexandrine parakeet](#_Toc475040066)

[Figure S3. Species distribution model for Ashy headed green pigeon](#_Toc475040067)

[Figure S4. Jackknife of regularized training gain for Ashy Headed Green Pigeon](#_Toc475040068)

[Figure S5. Species distribution model for Asian golden weaver](#_Toc475040069)

[Figure S6. Jackknife of regularized training gain for Asian Golden Weaver](#_Toc475040070)

[Figure S7. Species distribution model for Blossom headed parakeet](#_Toc475040071)

[Figure S8. Jackknife of regularized training gain for Blossom headed parakeet](#_Toc475040072)

[Figure S9. Species distribution model for White rumped pygmy falcon](#_Toc475040077)

[Figure S10. Jackknife of regularized training gain for White rumped pygmy falcon](#_Toc475040078)

Figure S11. Geo-locations of avian species

# Figure S1. Species distribution model for Alexandrine parakeet


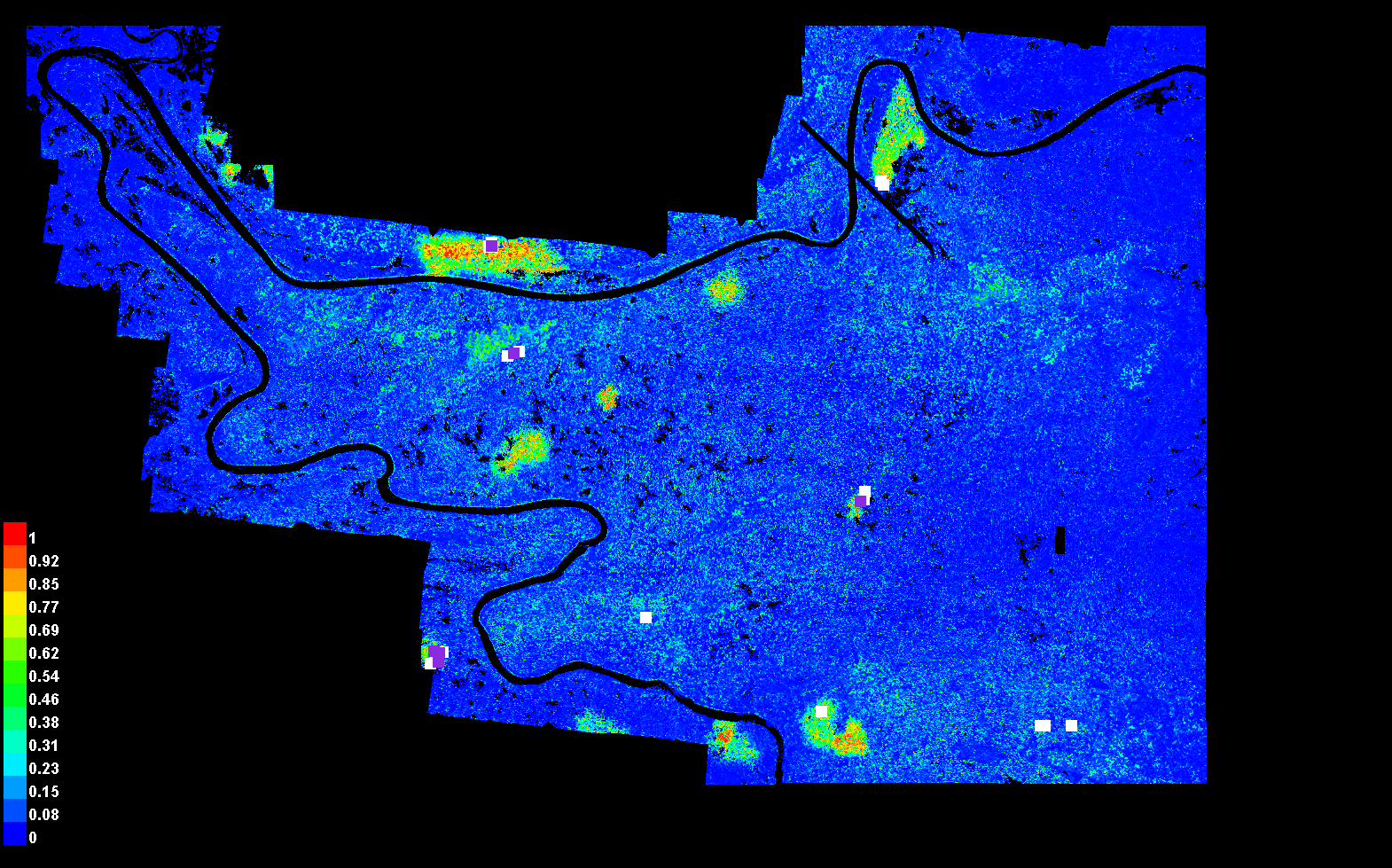


# Figure S2. Jackknife of regularized training gain for Alexandrine parakeet


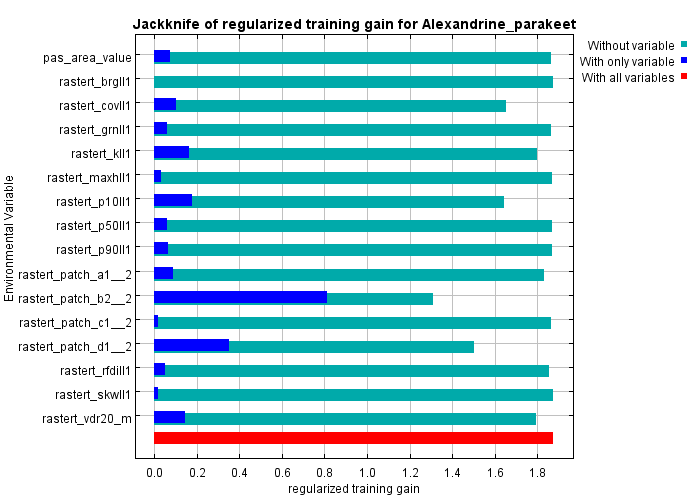


# Figure S3. Species distribution model for Ashy headed green pigeon


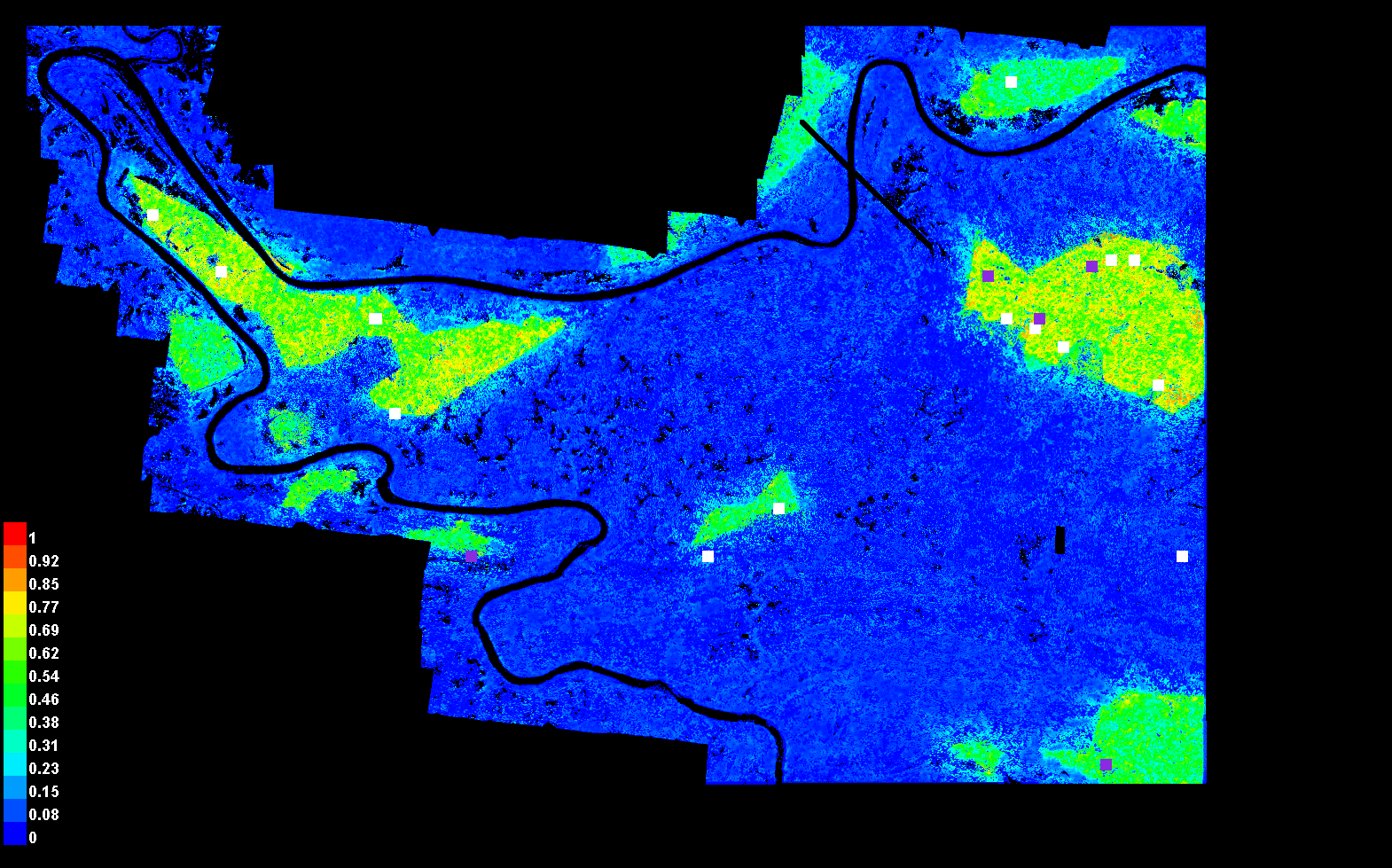


# Figure S4. Jackknife of regularized training gain for Ashy Headed Green Pigeon


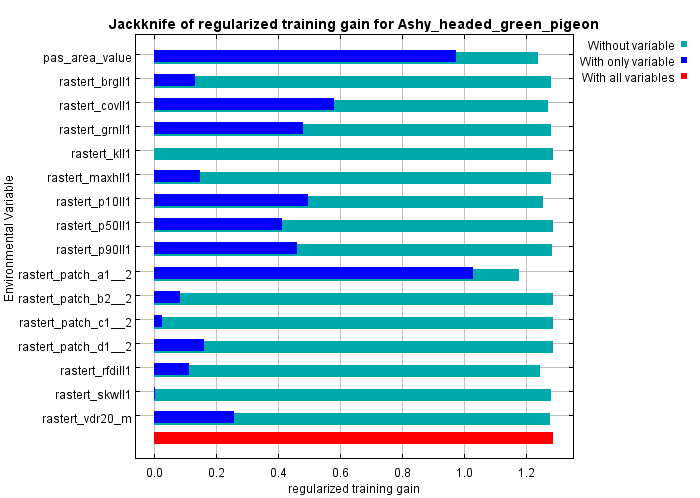


# Figure S5. Species distribution model for Asian golden weaver


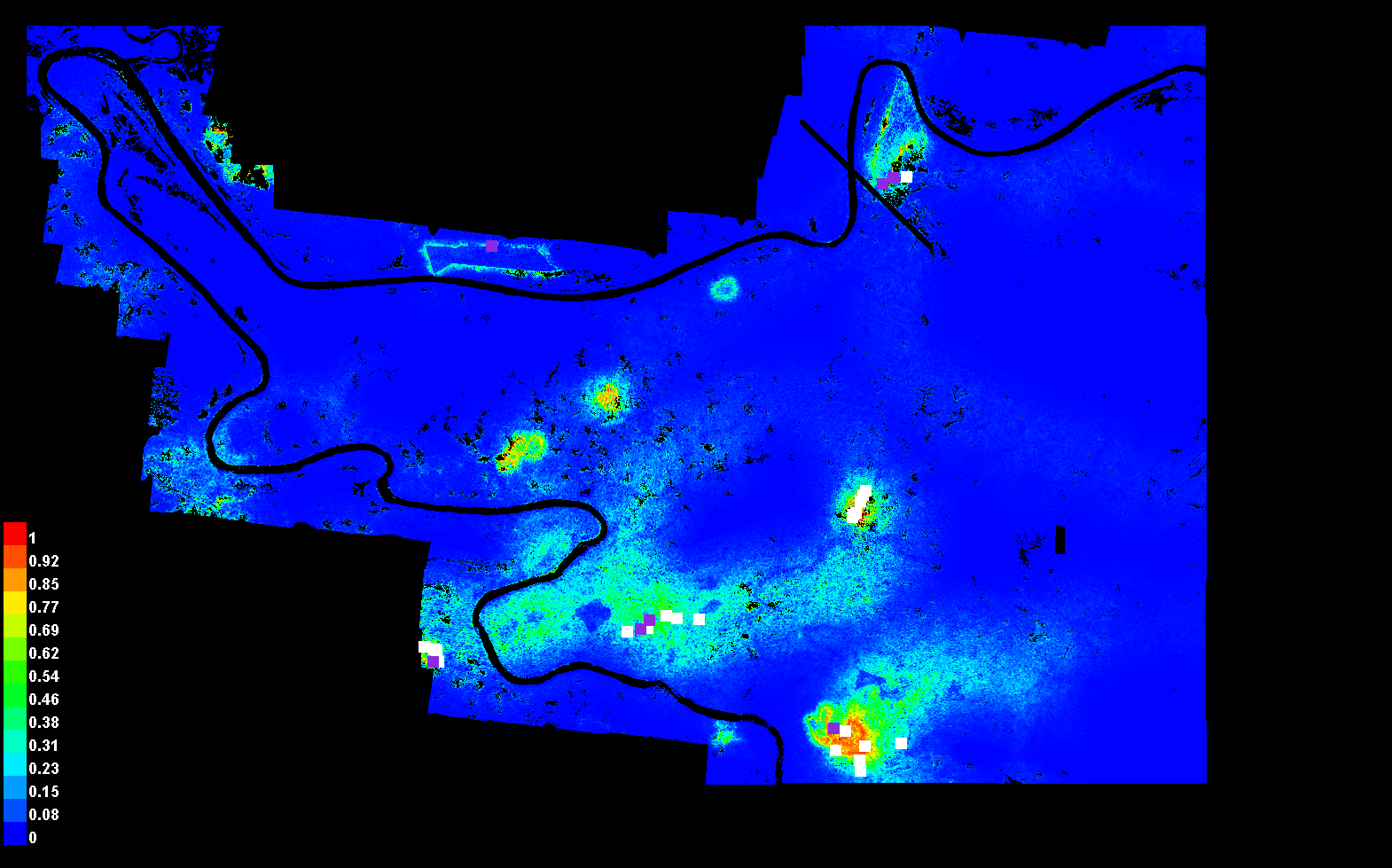


# Figure S6. Jackknife of regularized training gain for Asian Golden Weaver


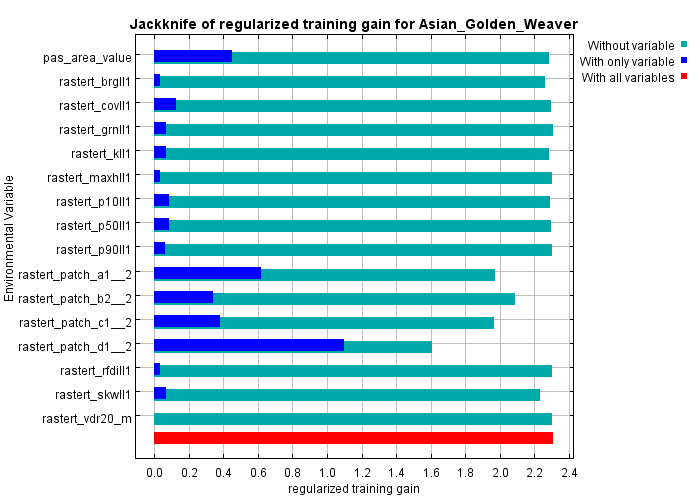


# Figure S7. Species distribution model for Blossom headed parakeet


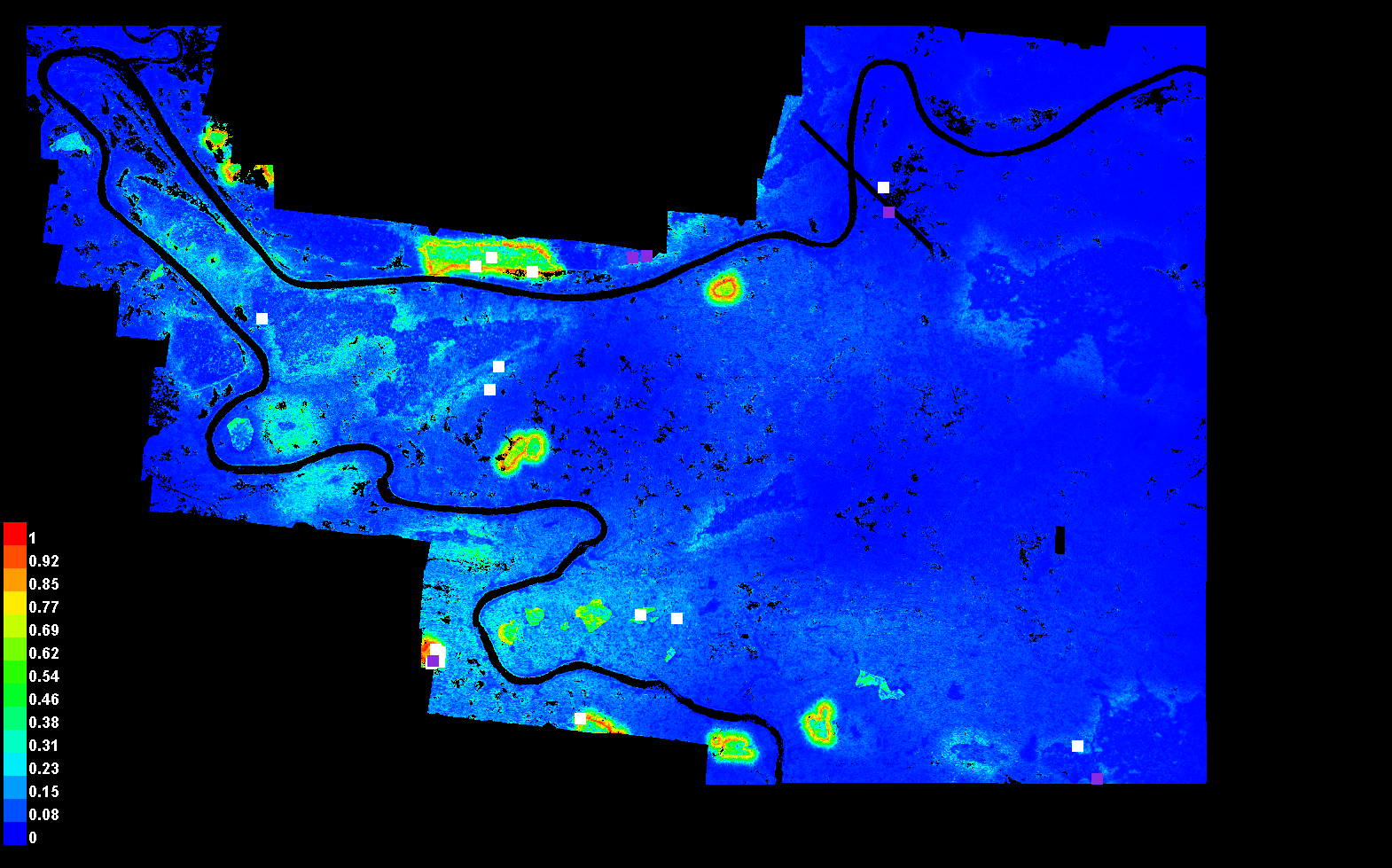


# Figure S8. Jackknife of regularized training gain for Blossom headed parakeet


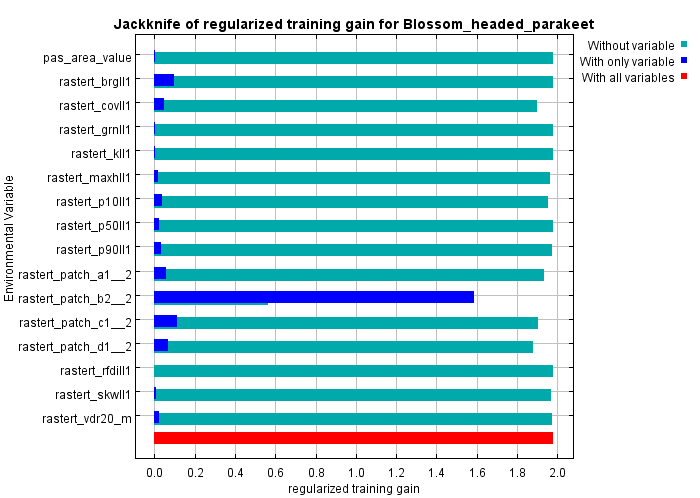


# Figure S9. Species distribution model for White rumped pygmy falcon


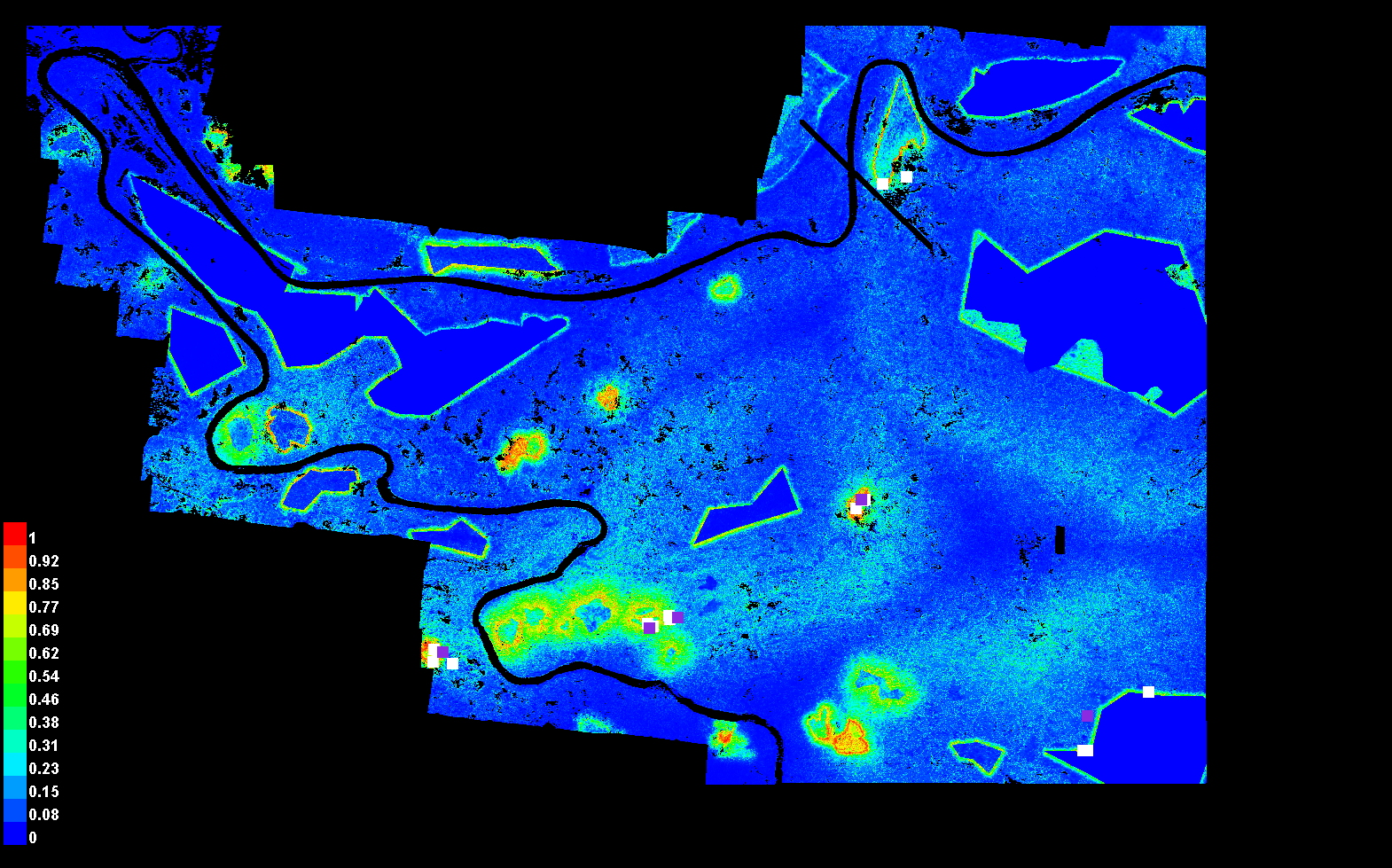


# Figure S10. Jackknife of regularized training gain for White rumped pygmy falcon


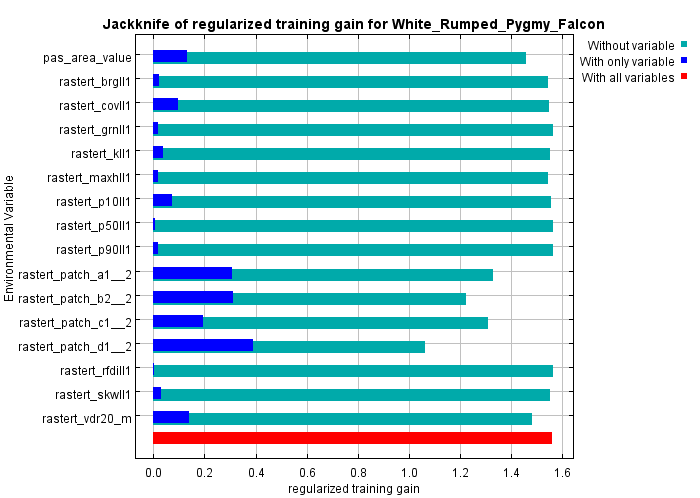


**S11 Figure. Target bird species geo-location map**

**
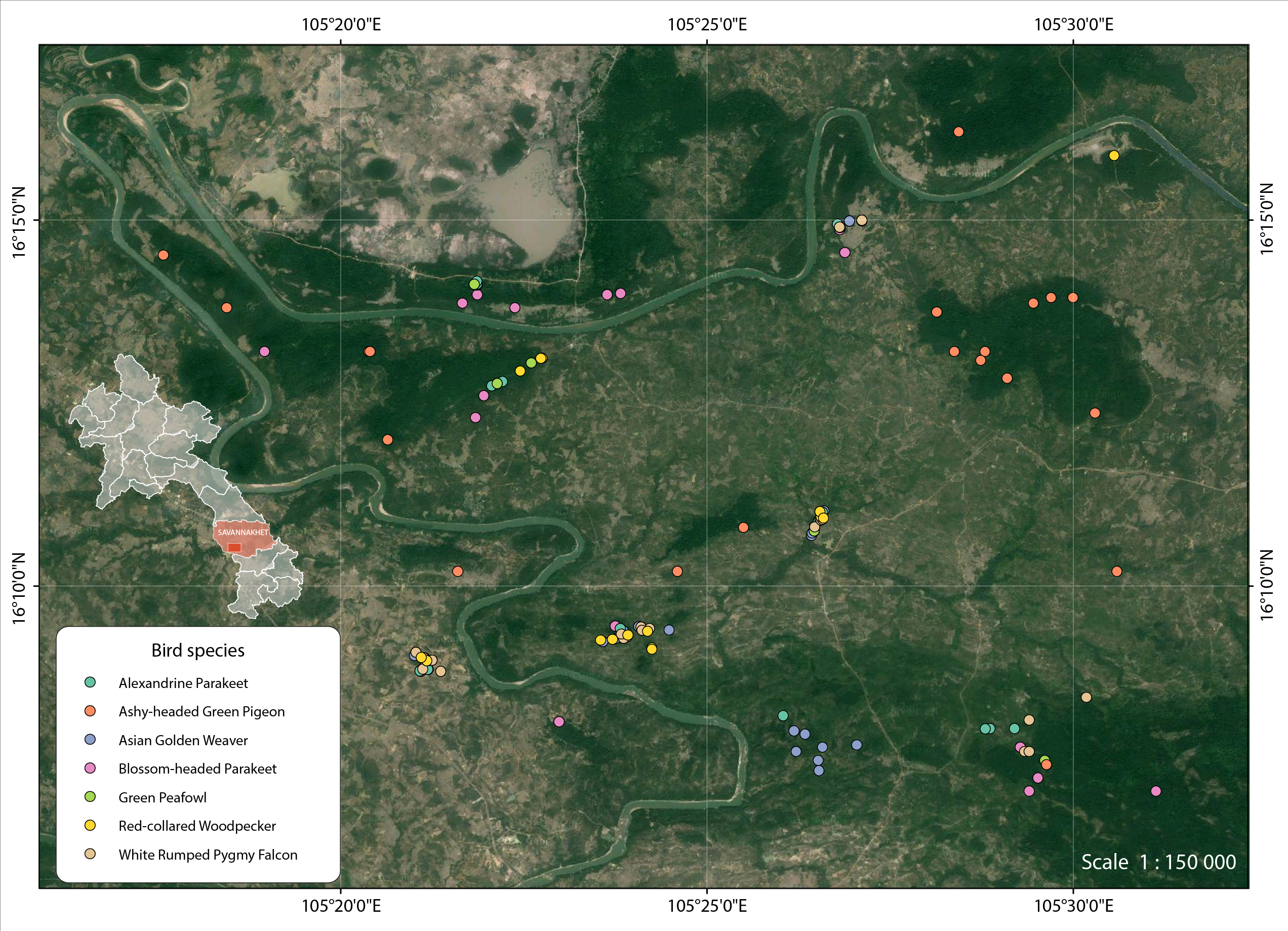
**
