# Supplementary figures and images for: Remote sensing‐based landscape indicators for the evaluation of threatened‐bird habitats in a tropical forest
Source: Ecol Evol. 2017 May 18;7(13):4552–67. doi: 10.1002/ece3.2970 (PMC5496523; doi:10.1002/ece3.2970)

**Figure 11. Target bird species geo-location map**

**
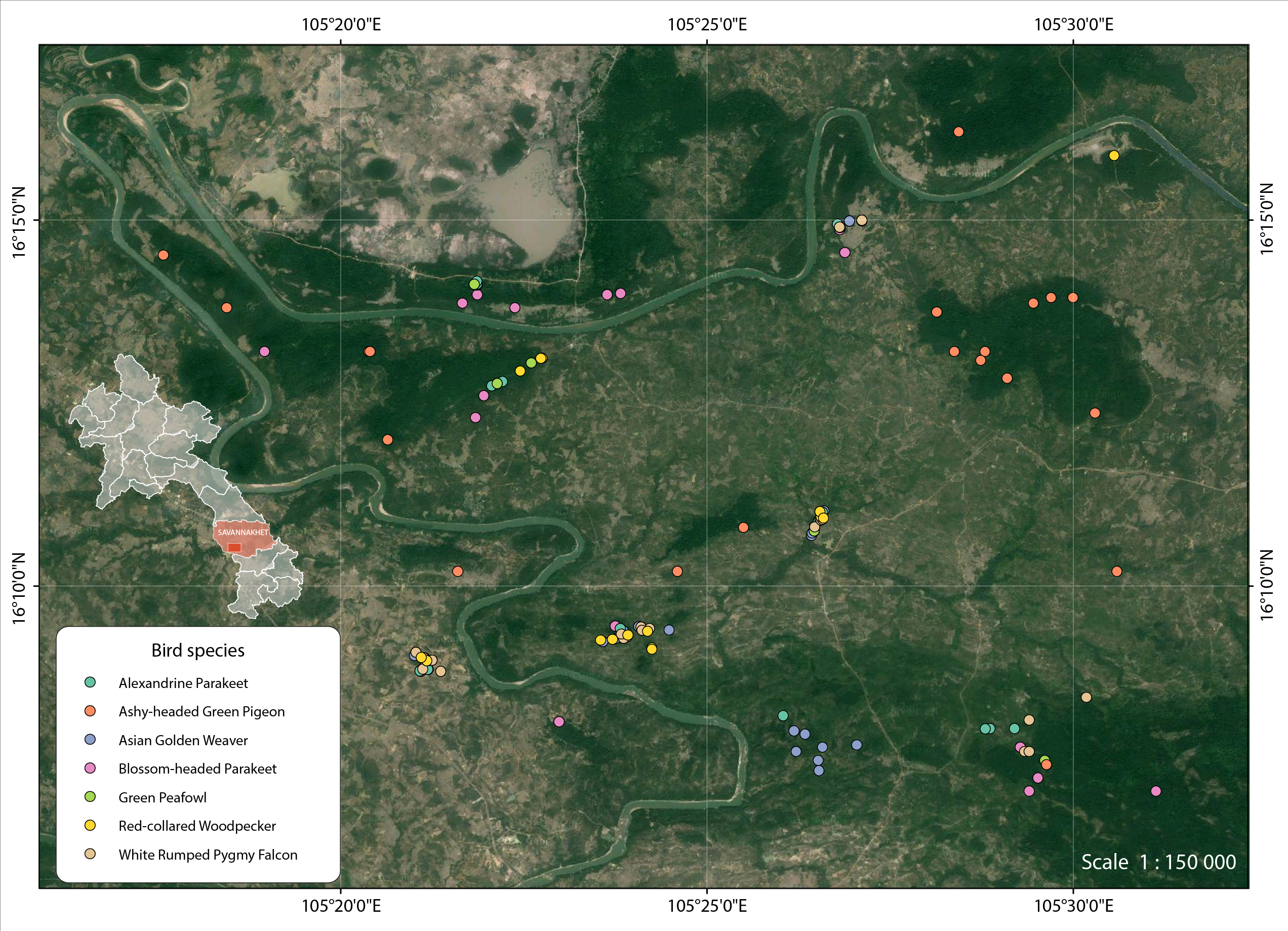
**

Supplement: Supplementary file 2 [file ECE3-7-4552-s002.docx]
